# Fact and Fiction Regarding Motorcycle Helmet Use, Associated Injuries, and Related Costs in the United States

**DOI:** 10.7759/cureus.3610

**Published:** 2018-11-19

**Authors:** Luke J Hofmann, Rachelle Babbitt-Jonas, Leen Khoury, Javier Martin Perez, Stephen M Cohn

**Affiliations:** 1 Surgery, University of Texas Health Science Center, San Antonio, USA; 2 Surgery, Staten Island University Hospital, Staten Island, USA; 3 Surgery, Hackensack Meridian Health, Hackensack, USA

**Keywords:** helmet, motorcycle crash, traumatic head injury

## Abstract

Background

Despite evidence that helmet use decreases motorcycle-associated injuries and mortality, the use of motorcycle helmets is not universal. As trauma surgeons are frequently the primary providers responsible for motorcycle crash victims, we sought to gain a better understanding of trauma surgeons’ perspectives on helmet use with motorcycles.

Methods

Members of the American Association for the Surgery of Trauma (AAST) were asked to participate in a survey that centered on attitudes, knowledge, and beliefs regarding motorcycle helmet use, associated injuries, and related costs. Demographic data were analyzed. In addition, we performed a literature search to attempt to clarify the current data on this subject.

Results

A total of 127 surgeons participated. The majority were male (64%, n=81), in academic practice (67%, n=85), and worked at a Level I trauma center (80%, n=102). Of those that owned a motorcycle, 100% wear a helmet when riding. Seven percent (n=9) of respondents believe helmet use increases cervical spine injury, although the majority (78%, n=99) disagree. In regards to head injuries and helmet use, most (93%, n=118) believe that helmets decrease the severity of head injury, improve outcomes (98%, n=124), and impact long-term disability (93%, n=118). Ninety percent (n=114) of surgeons believe that state legislation mandating motorcycle helmet use increases helmet utilization, and 82% (n=104) believe that the decision to wear a helmet should not be a personal decision. The majority (83%, n=106) of trauma surgeons agreed that helmet use would likely lead to a major reduction in motorcycle-related health care costs.

Conclusions

North American trauma surgeons wear helmets when they ride motorcycles and believe that these devices are highly protective, leading to a reduction in brain injury and the subsequent health care costs.

## Introduction

Although overall traffic-related fatalities have decreased [1], deaths associated with motorcycle use have increased by 55% since 2000 [2], and in 2010, more than 4,500 individuals died riding motorcycles [2-3]. According to government data, while only 20% of automobile crashes result in injury or death, the injury and/or fatality rate after motorcycle crashes approaches 80% [4]. The helmet is the most important piece of motorcycle protective equipment. In 2008, the federal government estimated that the use of helmets saved the lives of 1,829 motorcyclists, and if all motorcyclists had worn helmets that year, an additional 823 lives could have been saved [5]. Overall, the National Highway Traffic Safety Administration (NHTSA) estimates that helmets are 37% effective in preventing fatal injuries to motorcycle riders and 41% for motorcycle passengers [6]. Currently, only 19 states have mandatory universal motorcycle helmet laws in place, 28 states have partial helmet laws, and three states have no helmet laws at all [7]. This is a significant difference compared to the legislative requirements in 1975 when mandatory helmet laws were congressionally mandated. At that time, the Highway Safety Act linking federal highway construction funds to the presence of universal helmet laws led to the majority of states enacting universal helmet laws (only three states did not comply). After 1975, largely as a result of lobbying by motorcycle groups, Congress repealed the helmet requirement and associated penalties.

As the medical providers primarily responsible for the care and management of injured motorcyclists, trauma surgeons are uniquely impacted by the use or non-use of motorcycle helmets by their respective patient populations. As no definitive studies evaluating the benefits of helmet use are available, trauma surgeons’ opinions regarding this issue are important and could significantly impact legislative efforts to increase helmet use. We attempted to gain a better understanding of this highly controversial issue and conducted a survey to determine the current attitudes of trauma surgeons regarding motorcycle helmet use and associated outcomes.

## Materials and methods

After approval from the University of Texas Health Science Center at San Antonio Institutional Review Board, electronic surveys were sent in July 2013 to a contact list (email addresses) of all members of the American Association for the Surgery of Trauma (AAST). The email invitation included a brief description of the purpose of the survey along with a link to access the Survey MonkeyÒ site, an online survey program. On the Survey MonkeyÒ website, the first screen contained an information sheet asking the member if he or she agrees to participate in the voluntary survey. Respondents had the option of not participating or continuing in the survey at the bottom of the information screen.

For those participants who decided to participate, subsequent screens contained the survey questions. A five-point Likert scale was used to assess opinions on statements regarding helmet use and potential associated injuries and outcomes, scored as follows: 1= strongly agree, 2=agree, 3=neither agree nor disagree, 4=disagree, 5=strongly disagree. Identifying information was not collected on the Survey MonkeyÒ site (e.g., email address of the respondent).

A second email was sent two weeks after the initial invitation email encouraging members to complete the survey. The email specified that if the members had not previously completed the survey, they were being invited to do so. If the member had already completed the survey, they were asked to disregard the email. The survey was open for 30 days and locked out after this point.

We also endeavored to gather the data regarding essential facts about motorcycle helmet benefits and risks (Table 1).

**Table 1:** Basic Facts About Motorcycle Helmets

Basic Facts About Motorcycle Helmets
54% of Motorcycle Riders Do Not Wear Helmets [8].
41% of Motorcycle Riders Who Died in 2010 were Helmetless [9].
Deaths Associated with Motorcycle Use Has Increased by 55% since 2000 [2].
While only 20% of Automobile Crashes Result in Injury or Death, 80% of Motorcycle Crashes Lead to Injury or Death [4].
Helmets are 37% Effective in Preventing Fatal Injuries [6].
19 States and District of Columbia Have Universal Motorcycle Helmet Laws in 2018 [7].
28 States Have Some Helmet Laws [7].
3 States Have No Helmet Laws (Illinois, Iowa, and New Hampshire) [7].

## Results

Of the 750 members e-mailed, 127 (17%) participated and completed a survey. Ages ranged from 34 to 82, and the median age was 51. In stratifying the respondents into age groups, the majority were aged 49-66 (56%, n= 71), followed by a younger group aged 29-48 (38%, n=48), followed lastly by an older group aged 67-88 (6%, n=8). The majority of respondents had been in surgical practice for over 10 years (39% 11-20 years, n=49; and 39% over 20 years, n=50), with a minority in practice 0-10 years (22%, n=28). Most were male (64%, n=81) and worked at a Level I trauma center (80%, n=102). The most common practice setting was an academic medical center (67%, n=85), followed by a community medical center (20%, n=26).

The majority of respondents (92%, n=117) did not own a motorcycle. Of the individuals who owned a motorcycle, 40% (n=4) had been in a crash and all of them reported wearing a helmet. When asked whether the respondents’ state traffic regulations mandate the wearing of a helmet while operating a motorcycle, 39% (n=50) responded that helmets are required, 53% (n=67) responded that helmets are not required, and 8% (n=10) did not know.

When asked whether wearing a motorcycle helmet increases the likelihood of cervical spine injury, the majority (78%, n=99) of respondents disagreed although 7% (n=9) believed that helmet use increases the likelihood of cervical spine injury and 10% (n=13) neither agreed nor disagreed. In regards to helmet use decreasing the severity of head injuries after motorcycle crashes, the majority (93%, n=118) agreed with the statement and only (2%, n=3) disagreed.

When asked about outcomes associated with helmet use, 98% (n=124) stated that motorcycle helmets improve outcomes after motorcycle crashes and 93% (n=118) agreed that long-term disability is impacted by the use of helmets during crashes.

Most respondents (82%, n=104) do not believe that the decision to wear a helmet while operating a motorcycle should be a personal decision although 13% (n=17) feel that it should be a personal decision. The majority of respondents (90%, n=114) believe that state legislation mandating motorcycle helmet use increases helmet use and 84% (n=107) believe that all states should require motorcycle helmets be worn during operation.

The majority of respondents (70%, n=89) disagreed that insurance dollars would typically cover almost all of the medical costs associated with motorcycle crashes, 17% (n=22) neither agreed nor disagreed, and 13% (n=16) agreed. Most (83%, n=106) respondents agreed that helmets would likely lead to a major reduction in the health care costs associated with motorcycle crashes, but 8% (n=10) neither agreed nor disagreed and another 8% (n=10) disagreed with the statement.

## Discussion

In our survey, 100% of the trauma surgeon respondents who owned a motorcycle reported wearing a helmet when riding. This is significantly higher than the estimated 54% of U.S. motorcycle riders who wear a helmet [8]. Although the frequency of voluntary helmet use among motorcycle operators is not known, 41% of motorcycle operators and 50% of motorcycle passengers who died in 2010 were not wearing a helmet [9].

Motorcycle helmet laws vary widely among the states. As of October 2018, 19 states and the District of Columbia have laws requiring all riders (operators and passengers) to wear a helmet, known as universal helmet laws. Laws requiring only some motorcyclists to wear a helmet are in place in 28 states. There is no motorcycle helmet use law in three states (Illinois, Iowa, and New Hampshire) [7].

Nearly 80% of respondents felt that helmets were beneficial in preventing cervical spine injury. However, the Cochrane review in 2008 concluded that “there is insufficient evidence to make conclusions about helmet effects on neck and facial injuries” [10]. More recent studies, however, suggest that helmets may be protective. A retrospective analysis of 713 motorcycle injuries concluded that helmets reduced the risk of cervical spine injury by at least 50% [11]. Similarly, a large National Trauma Data Bank (NTDB) review of 62,480 motorcycle collisions found that helmeted riders had a lower proportion of cervical spine injury (3.5% vs 4.4%) as compared with nonhelmeted riders [12].

When asked whether helmets decrease the severity of head injuries, a larger majority (93%, n=118) of surgeon respondents agreed. Similarly, a significant majority (98%, n=124) felt that motorcycle helmets improve outcomes after motorcycle crashes. This is consistent with the published literature since numerous studies, including a Cochrane review, suggest that motorcycle helmets reduce both mortality and traumatic brain injuries. As stated in the Cochrane review discussion, “…there is no doubt that motorcycle helmets compared with no helmets reduce the likelihood of head injuries” [10]. Additionally, they determined that “studies estimating the effect of helmets on mortality were very consistent in their results, suggesting a protective effect.” The National Highway Traffic Safety Administration has also concluded that helmets reduce traumatic brain injury by 67% and mortality by 35% [13].

The majority of respondents (82%, n=104) do not believe that the decision to wear a helmet while operating a motorcycle should be a personal decision, and 84% (n=107) believe that all states should require motorcycle helmets to be worn during operation. In addition, 90% (n=114) of respondents believe that state legislation requiring motorcycle helmets be worn increases helmet use. It does appear that the existence of a helmet law affects helmet use. According to a 2010 survey from the National Highway Traffic Safety Administration, compliance in wearing helmets in states where use is required for all motorcyclists was 86% versus 55% in states where it was not [8]. Similarly, in a retrospective review of motorcycle collisions in the National Trauma Data Bank, helmets were worn in 90% of motorcycle collisions from states with universal laws [14].

Only a minority of respondents (13%, n=16) agreed that insurance dollars would typically cover almost all of the medical costs associated with motorcycle crashes; 70% disagreed with the statement. Most respondents (83%, n=106) agreed that helmets would likely lead to a major reduction in motorcycle-related health care costs. It is estimated that the economic burden of injuries and deaths from motorcycle-related crashes in one year totaled $12 billion [15]. Hospital charges for riders wearing helmets are significantly less than charges for those not wearing helmets ($4184.26 versus $7383.31). In addition, Intensive Care Unit (ICU) Length of Stay (LOS), hospital LOS, and ventilator days are of statistically significant longer duration for unhelmeted patients [16].

Similarly, in a large study of 104,472 motorcycle crashes using the National Highway Traffic Safety Administration (NHTSA) Crash Outcome Data Evaluation System (CODES), unhelmeted motorcycle riders injured in a crash and admitted to hospitals faced substantially higher healthcare costs than helmeted riders, primarily due to the higher incidence and severity of traumatic brain injury [17]. The differential healthcare economic burden between unhelmeted and helmeted motorcyclists has been estimated to be about $250,231,734 per year [18].

Who pays for the costs associated with motorcycle injuries? A study of 105 motorcyclists hospitalized at a major trauma center found that 63% of their care was paid for by public funds, with Medicaid accounting for over half of all charges [19].

Unhelmeted motorcycle riders are also less likely to have health insurance and, therefore, more likely to have their medical expenses paid by government-funded healthcare [20]. Therefore, it appears that not only is the care of unhelmeted motorcyclists more expensive, but it comes at a public cost as well. Unfortunately, the only safety measure that has been proven to improve motorcycle safety is the institution of state universal helmet laws [21]. It is also a measure that is inexpensive and impacts all motorcycle riders (Table 2).

**Table 2:** Controversies Around Motorcycle Helmets

Controversies Around Motorcycle Helmets
Question One: Are Cervical Spine Injuries Reduced By Helmets?
Answer: Yes, Helmets appear to reduce cervical spine injuries by 50% [9].
Question Two: Do Helmets Reduce the Incidence of Traumatic Brain Injury (TBI)?
Answer: Yes, Helmets reduce TBI by about 67% [11].
Question Three: Do Helmets Save Lives?
Answer: Yes, Helmets reduce mortality by 35% [11].
Question Four: Do Insurance Dollars Cover Medical Care After Motorcycle Crashes?
Answer: No, Most (2/3) of the >$12 billion yearly cost of medical care after motorcycle crashes is borne by the public sector [18].
Question Five: Should Helmet Wearing Be a Personal Decision?
Answer: No, Helmet laws are beneficial and compliance is excellent where regulations are universal [13].

## Conclusions

We concluded that North American trauma surgeons wear helmets when they ride motorcycles and believe that these devices are highly protective, leading to a reduction in brain injury and subsequent health care costs. There appear to be ample data to address many of the areas of controversy surrounding the risks and benefits of motorcycle helmet use.
